# Clinical Use Patterns and Patient-Reported Experience with Inhaled Levodopa in Routine Practice: A Two-Center Study

**DOI:** 10.3390/medsci14040446

**Published:** 2026-07-28

**Authors:** Tania Delgado Ballestero, Sonia Escalante Arroyo, Marcos Alfredo Mandrá Noya

**Affiliations:** 1 Neurology Department, Consorci Corporació Sanitària Parc Taulí, Hospital Universitari Parc Taulí, 08208 Sabadell, Spain; 2 Neurology Department, Hospital Universitari de Tortosa Verge de la Cinta, Institut Català de la Salut, 43500 Tortosa, Spain; sescalante.ebre.ics@gencat.cat (S.E.A.); malfredo.ebre.ics@gencat.cat (M.A.M.N.); 3 Institut d’Investigació Sanitària Pere Virgili (IISPV), 43005 Tarragona, Spain

**Keywords:** Parkinson’s disease, patient experience, fluctuations, inhaled levodopa, on-demand therapies, satisfaction

## Abstract

**Background/Objectives**: On-demand therapies are crucial to manage OFF episodes in Parkinson’s disease (PD). Inhaled levodopa is a rapid and non-invasive treatment option, but data on its real-world use and patient-reported experience remain limited. This study characterized clinical use patterns and patient experience with inhaled levodopa in routine clinical practice. **Methods**: We conducted a multicenter, cross-sectional, observational study including patients with PD treated with inhaled levodopa in two Spanish centers. Data on treatment use, patient-reported clinical response, and satisfaction were collected during a single study visit using study-specific questionnaires. **Results**: Thirty-five patients were included (mean age 70.6 years; 51.4% women). Most patients used inhaled levodopa once daily (51.4%), with administration patterns varying between on-demand (51.4%), fixed (31.4%), and combined regimens (17.1%). According to routine clinical assessments, 80.0% of patients reported a clinical response to inhaled levodopa, with a mean time to onset of 17.7 min and a mean duration of effect of 122.1 min. The perceived response was rated as moderate or marked in 62.8% of patients and was reported to last until the next scheduled oral levodopa dose in 45.7% of patients. Participants highlighted symptom improvement, ease of identifying the appropriate timing of administration, and a favorable perception of treatment safety. **Conclusions**: In routine clinical practice, inhaled levodopa was associated with a high patient-reported clinical response and favorable treatment experience across diverse patient profiles. These findings provide relevant insights into the real-world use of inhaled levodopa as an on-demand therapy for OFF episodes in PD. Given the exploratory nature of this study, confirmation in larger prospective studies using validated instruments and objective clinical measures is warranted.

## Simple Summary

People with Parkinson’s disease often experience periods when their usual medication stops working and symptoms suddenly return. These episodes can interfere with daily activities, reduce independence, and increase anxiety. Inhaled levodopa is a fast-acting treatment designed to relieve these episodes, but there is limited information about how patients use it and experience it in everyday clinical practice. We studied 35 people with Parkinson’s disease treated at two hospitals in Spain and asked them about their experience with inhaled levodopa. Most participants reported that the treatment improved their symptoms, worked quickly, and was easy to use. Many also found it simple to identify the right moment to take it and considered it practical for daily life. The treatment was generally well tolerated, and patients with different clinical characteristics reported similar experiences. These findings provide real-world information about how inhaled levodopa is used outside clinical trials and suggest that it may be a useful option for managing sudden symptom worsening in people with Parkinson’s disease.

## 1. Introduction

Parkinson’s disease (PD) is a neurodegenerative disorder characterized by the presence of both motor (bradykinesia, postural instability, rigidity, and tremor) and non-motor (cognitive impairment, sleep disorders, gastrointestinal or genitourinary dysfunction, among others) symptoms [1]. Levodopa remains the gold-standard treatment for PD due to its high efficacy in controlling symptoms [2]. However, after an initial period of marked improvement, its clinical efficacy declines as the disease progresses [3], leading to the development of chronic treatment-related complications such as motor and non-motor fluctuations and dyskinesias [4].

Clinical fluctuations may affect approximately 40% of patients after 4–6 years of treatment [5]. They are characterized by alternating periods of symptom improvement (“ON” periods) and periods of reduced or suboptimal response during which symptoms re-emerge or worsen (“OFF” periods) [3]. OFF episodes occur as a consequence of neurodegeneration, pulsatile dopaminergic stimulation, delayed gastric emptying, and interference with dietary proteins. Although OFF episodes typically develop progressively, they may also occur unpredictably, thereby impairing patients’ autonomy and quality of life [6].

The management of motor fluctuations is based on adjusting the levodopa dose and dosing frequency; however, this strategy has limitations and may even worsen OFF periods [7]. Alternative therapeutic options, such as dopamine agonists, catechol-O-methyltransferase (COMT) inhibitors, or monoamine oxidase B (MAO-B) inhibitors, may reduce OFF time, although the reduction is frequently only partial [8]. Consequently, many patients continue to experience residual and unpredictable OFF episodes that substantially limit daily functioning and quality of life [9]. This highlights the need for on-demand therapies with a rapid, predictable, and reliable onset of action and that are easy to administer. Such therapies may be considered before or alongside device-aided therapies, depending on the patient’s needs [10]. Currently, three treatments are approved for the management of OFF episodes: subcutaneous apomorphine, sublingual apomorphine, and inhaled levodopa [10].

Among these options, inhaled levodopa (Inbrija^®^) offers the advantage of using the gold-standard treatment for PD without introducing a new therapeutic class and requiring dose titration [6]. By bypassing the gastrointestinal tract, it achieves faster, more consistent, and more predictable absorption than oral levodopa formulations [9]. Furthermore, the pulmonary administration enables high plasma concentrations to be reached within minutes, resulting in a rapid clinical onset of action. Several clinical trials have demonstrated its rapid onset of action, significant motor improvements, and favorable safety profile [11,12,13,14].

Despite the available evidence from clinical trials, information regarding the use and patients’ experiences with inhaled levodopa in routine clinical practice remains limited. This aspect is particularly relevant in the context of on-demand therapies, as factors such as ease of use, identification of the appropriate time of administration, and the perception of symptom control may significantly influence their clinical use.

The aim of this study was to describe patients’ clinical experiences and satisfaction with inhaled levodopa in routine clinical practice.

## 2. Materials and Methods

### 2.1. Study Design

This multicenter, cross-sectional, observational study was conducted at two Spanish centers. The study was conducted in accordance with the Declaration of Helsinki, and the protocol was approved by the Research Ethics Committees of both participating centers. Informed consent was obtained from all subjects involved in the study.

The study consisted of a single visit, during which patients or caregivers provided information on inhaled levodopa use and completed global treatment experience and satisfaction questionnaires. All questionnaires were completed by the patients. In participants with dementia-stage cognitive impairment, questionnaire responses were reviewed and confirmed by their primary caregivers. Clinical and sociodemographic data were obtained from medical records.

### 2.2. Study Population

Between December 2024 and May 2025, all patients who had been prescribed inhaled levodopa at the two participating centers were screened for eligibility. Patients were included if they had sufficient experience using inhaled levodopa to complete the study questionnaires and provided written informed consent. No eligible patient declined participation. Patients were excluded if they had never used inhaled levodopa, were unable to use the inhaler because of technical difficulties and therefore had no treatment experience on which to base the questionnaire responses or had used inhaled levodopa more than 6 months before the study visit and were unable to reliably recall their treatment experience. Patients with dementia-stage cognitive impairment were excluded unless they had a regular caregiver.

### 2.3. Study Outcomes

The objective of the study was to describe the clinical experience with inhaled levodopa for the treatment of OFF periods in patients with PD in routine clinical practice. To this end, we collected clinical data, patient-reported clinical response, patterns of inhaled levodopa use, duration of response, and the presence of treatment-related adverse events. PD stage was assessed using the Motor/Non-motor/Cognition/Dependency (MNCD) classification tool [15]. Additionally, patients completed two purpose-made questionnaires: the global experience and the satisfaction questionnaire.

As no validated instrument was available to specifically assess the real-world use and patient experience with inhaled levodopa in routine clinical practice, the study investigators developed two purpose-specific questionnaires for this study. The questionnaire items were selected by consensus among the investigators. The instruments were intended as exploratory descriptive tools and were not formally validated before use. The global experience questionnaire consisted of four domains (efficacy, ease of use, side effects, and impact on activities of daily living), each comprising four items. Each question was scored as 1 or 0 depending on the presence or absence of the characteristic assessed. Results are expressed as the percentage of patients with a positive response for each item. The global experience questionnaire can be found in Supplementary Table S1. Information on adverse events was obtained from medical record review, whereas the global experience questionnaire evaluated the perceived impact of these adverse events on the patients’ overall treatment experience.

Treatment satisfaction with inhaled levodopa was assessed using a 5-point Likert scale. Response options were 1 = not at all satisfied; 2 = slightly satisfied; 3 = neutral (neither satisfied nor dissatisfied); 4 = quite satisfied; and 5 = very satisfied. Treatment satisfaction is expressed as the number and proportion of patients in each response category.

The exploratory objectives of the study were to characterize patterns of inhaled levodopa use and to explore factors that may influence patient-reported response and satisfaction. To this end, these variables were assessed according to patients’ cognitive impairment, physical activity level, levodopa administration regimen (fixed, on-demand, or combined), and dependency status. Cognitive impairment was determined according to clinical judgment, based on the available clinical information, including cognitive complaints reported by the patient and/or caregiver and, when available, the results of standardized cognitive assessments. Patients were considered sedentary if they walked less than 30 min per day [15]. Dependency was classified according to the dependency domain of the MNCD scale as: independent for activities of daily living (score 0), dependent only for instrumental activities of daily living (score 1), or dependent for basic activities of daily living (score 2) [16].

### 2.4. Statistical Analysis

Statistical analyses were performed using the SAS software for Windows, version 9.4. A descriptive analysis of the study variables was conducted. Continuous variables were described using the mean, standard deviation (SD), and median, whereas categorical variables were summarized as frequencies and percentages. To assess whether the variables of interest differed across patient subgroups, patients were stratified into different groups.

## 3. Results

### 3.1. Study Population

**Figure 1:**
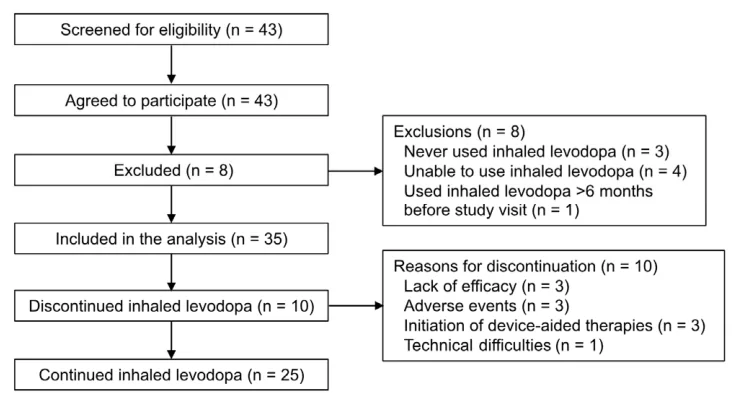
Patient disposition.

Of the 43 patients screened for eligibility, all agreed to participate. Eight were subsequently excluded: 3 had never used inhaled levodopa, 4 were unable to use the inhaler due to technical difficulties, and 1 had used inhaled levodopa more than 6 months before the study visit. A total of 35 patients (51.4% women) were included. Regarding treatment persistence, 10 of 35 patients discontinued inhaled levodopa. The reasons for discontinuation were lack of efficacy (n = 3), technical difficulties using the inhaler that prevented effective treatment administration (n = 1), adverse events (n = 3), and initiation of device-aided therapies (n = 3). Discontinuation due to adverse events occurred in 3 of 35 patients (8.6%) (Figure 1). There were no missing data for patient-reported responses, questionnaire items, adverse event reporting, or subgroup variables.

The mean age in the overall population was 70.6 years and the mean duration of PD was 10.3 years. Twenty patients (57.1%) had cognitive impairment, 17 (48.6%) mild and 3 (8.6%) classified as dementia. The mean duration of motor fluctuations was 3.9 years. The main indications for the use of inhaled levodopa were end-of-dose deterioration (68.6%) and morning akinesia (57.1%). The mean levodopa equivalent daily dose (LEDD) was 1020.4 mg (Table 1).

### 3.2. Inhaled Levodopa Treatment

Among the study population, 51.4% of patients received a single daily dose of inhaled levodopa, while 17.1% received two daily doses. Inhaled levodopa was administered on demand in 51.4% of cases, according to a fixed dosing schedule in 31.4%, and as part of a combined regimen in 17.1%.

According to routine clinical assessments, 28 of 35 patients (80.0%) reported a clinical response to inhaled levodopa, with a mean response duration of 122.1 min (median: 90.0 min) and a mean time to response of 17.7 min (median: 15.0 min) (Table 1). The response was moderate in 31.4% of cases, marked in 31.4%, and mild in 17.1%. In 45.7% of cases, the duration of response lasted until the next scheduled oral levodopa dose (Table 2). Overall, 25 of 35 patients (71.4%) continued treatment.

### 3.3. Global Experience

In the patient-reported global experience questionnaire, 26 of 35 patients (74.3%) reported an improvement in symptoms after using inhaled levodopa. In addition, 65.7% reported that the dose was sufficient to control symptoms and that its effect lasted until the next scheduled dose.

With respect to ease of use, 77.1% reported that it was easy to identify the appropriate time for administration, while 71.4% reported being able to administer the dose without assistance and considered the device practical and convenient to carry.

In terms of tolerability, 8.6% of participants reported neurological side effects and 34.4% reported respiratory side effects. Overall, 17.1% reported that these adverse effects affected treatment adherence.

**Figure 2:**
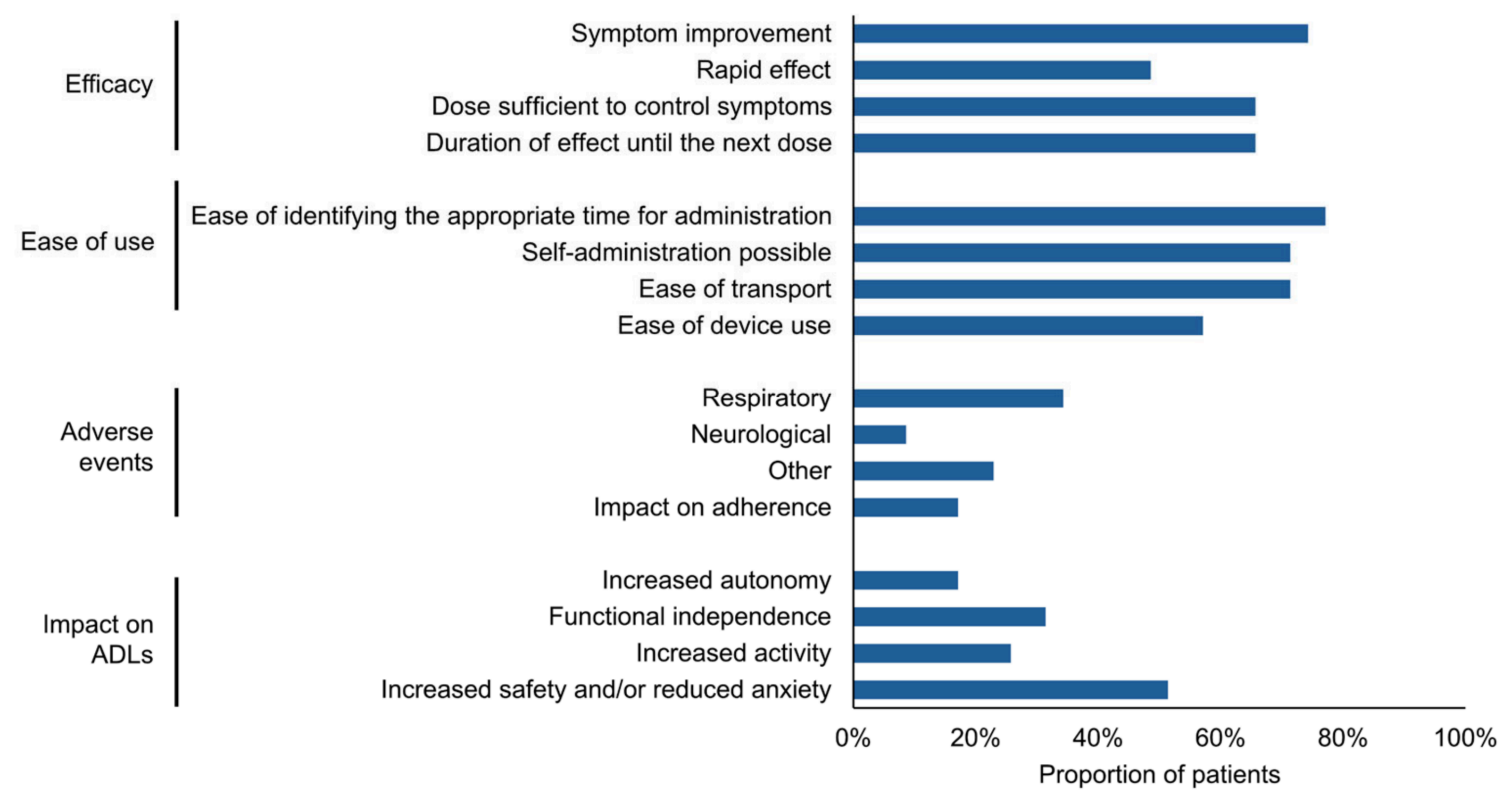
Patient-reported treatment experience. ADLs, Activities of Daily Living.

Regarding the impact on activities of daily living, 51.4% reported feeling safer and/or less anxious with the treatment, whereas the remaining items related to this domain obtained proportions below 50% (Figure 2).

### 3.4. Satisfaction

The proportion of patients who reported being satisfied/very satisfied with inhaled levodopa was 48.5% (37.1% quite satisfied and 11.4% very satisfied), while 22.9% reported feeling neutral (neither satisfied nor dissatisfied).

### 3.5. Adverse Events

Twenty-one adverse events were reported in 16 (45.7%) patients, of which cough was the most frequent (10 events) (Table 3).

### 3.6. Factors Associated with Inhaled Levodopa Use

**Figure 3:**
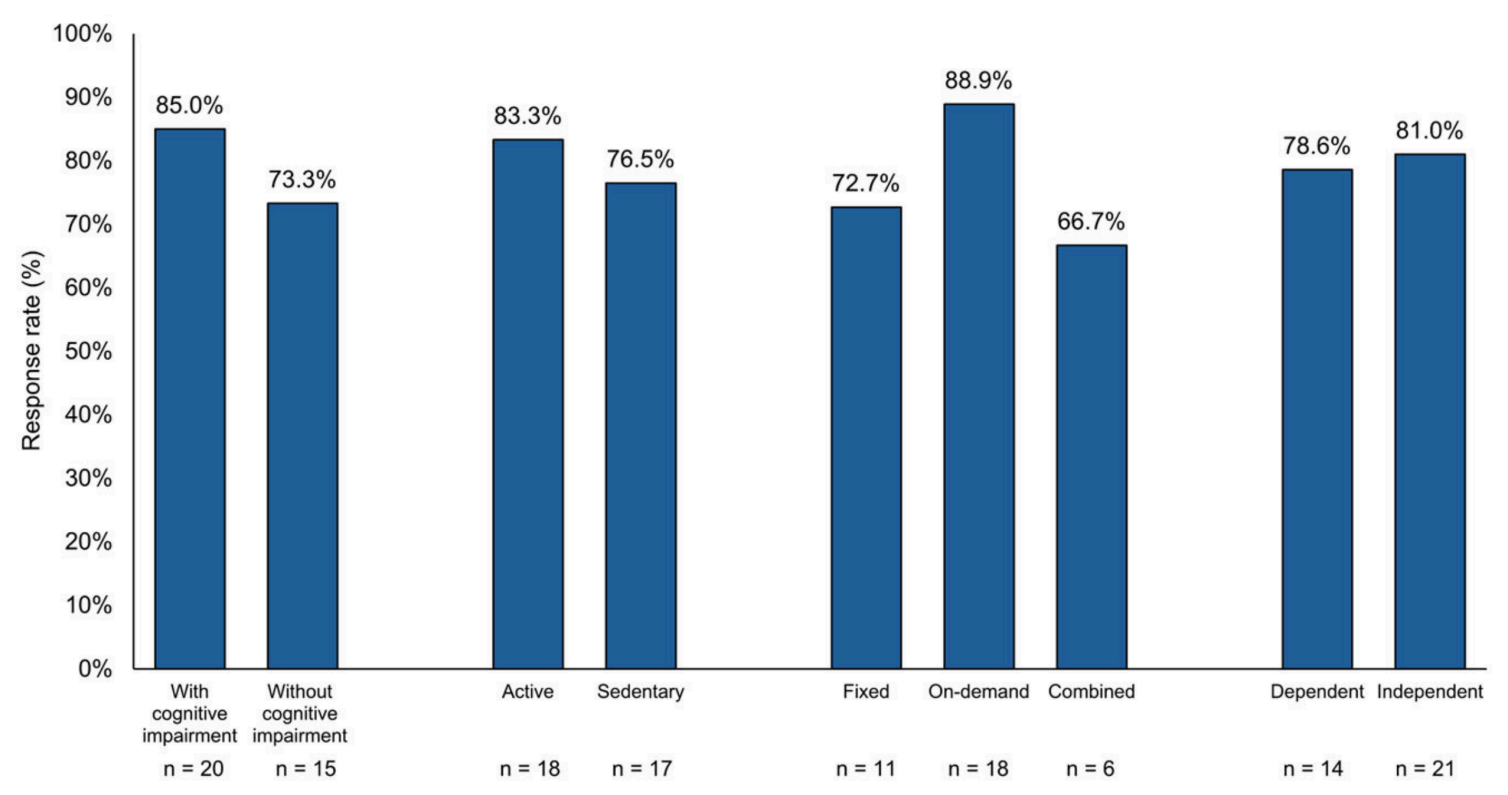
Factors associated with inhaled levodopa response.

Similar patient-reported response rates, time to response and degree of response were observed regardless of the presence of cognitive impairment, activity levels, levodopa administration regimen, or dependence status (Figure 3, Supplementary Table S2).

Comparable scores were observed across all domains of the patient global experience questionnaire regardless of the presence of cognitive impairment. Similarly, satisfaction with inhaled levodopa was comparable regardless of cognitive impairment, activity level, administration modality, or dependency status.

## 4. Discussion

In this study, we explored the experiences of patients with PD and motor fluctuations treated with inhaled levodopa in routine clinical practice. The results showed that most patients perceived symptom relief after inhalation and that the treatment was well accepted and easy to use, particularly among those who were able to self-administer the dose or followed an on-demand regimen.

The treatment of motor fluctuations in PD continues to represent a clinical challenge, not only because of the unpredictable occurrence of OFF episodes, but also because of predictable OFF episodes that do not respond adequately to oral treatment. Both situations can be highly disruptive and cause considerable stress in patients’ daily lives [9]. In this context, on-demand inhaled therapies offer relevant advantages [17,18] and are considered effective for the treatment of OFF periods that do not respond adequately to oral therapy [2].

However, because of their relatively recent introduction, clinical practice guidelines still provide limited recommendations, which may contribute to the underuse of these therapies [19,20]. In this context, observational studies incorporating the patient perspective provide essential information on how on-demand therapies are used, the challenges encountered, and the characteristics most valued by patients [9].

In our cohort of 35 patients, the use of inhaled levodopa was less frequent than that reported in clinical trials. Although the prescribing information indicates a maximum of five inhalations per day [21], half of the patients used it only once daily and 17.1% twice daily, with a mean of 1.3 administrations per day, compared with 2.1 administrations reported in a phase 2b study [12]. Most patients used it on demand (51.4%), while 31.4% followed a fixed regimen. Recent studies suggest that administration at the onset of OFF symptoms optimizes the therapeutic response; however, some patients with predictable fluctuation patterns prefer physicians to establish a fixed regimen, possibly due to concerns about self-medication [6]. In our series, most patients following a fixed regimen used inhaled levodopa for the management of morning akinesia, achieving a good clinical response, consistent with previous studies [22].

Notably, 80% of patients reported a clinical response, with a mean time to response of 17.7 min and a mean response duration of 122.1 min, persisting until the next scheduled oral levodopa dose in 45.7% of patients. Although these findings are based on patient-reported clinical responses, they are consistent with previous evidence showing an onset of effect within 5–10 min, a Tmax at 15 min, and peak effect at 30 min, with a dose-dependent response [12,17,22,23]. The rapid onset of action and prolonged duration of effect are particularly relevant, as reflected in a discrete choice experiment in which participants expressed a preference for treatments with a rapid onset and prolonged duration (2 h) for the management of OFF episodes [19]. In this context, previous studies have also demonstrated that inhaled levodopa absorption is faster and more consistent compared with oral levodopa for the treatment of OFF episodes, and is associated with more rapid improvement in motor function [24].

The results of the global experience questionnaire reinforce these findings, with most patients reporting sustained improvement until the next scheduled oral levodopa dose. These real-world patient-reported findings complement those from phase 2b and phase 3 trials, which demonstrated significant motor improvements in the Unified Parkinson’s Disease Rating Scale part III (UPDRS-III) [12,13], as well as with a long-term study reporting that more than 80% of subjects achieved an ON state within the first 60 min [14].

The features of inhaled levodopa most valued by patients regarding ease of use were the ability to self-administer the treatment and the convenience of carrying the device. This is consistent with findings from clinical trials, which reported short self-administration times (1.5–1.9 min), and the only difficulty reported by more than one patient was perforating the capsules [9]. Although an initial demonstration of the device is recommended, unlike subcutaneous and sublingual apomorphine, the first administration of inhaled levodopa does not require medical supervision [6,20].

The improvements in activities of daily living were modest, although half of the patients reported feeling safer and/or less anxious with inhaled levodopa. This finding is consistent with the multidimensional nature of this variable and may be explained by the fact that the specific benefit of rescue therapy may not necessarily translate into substantial improvements in daily activities. However, the sense of control over OFF episodes may substantially reduce anxiety associated with unpredictability and inadequate response to oral levodopa, both of which often limit autonomy and social participation [6,9].

Regarding safety, adverse events were frequent, although mild in most patients, with cough being the most common. Treatment discontinuation due to adverse events occurred in only 8.6% of patients and, in some cases, adverse events led to a reduction in administration frequency. Consistent with these findings, cough reported in clinical trials of inhaled levodopa was predominantly mild to moderate and typically occurred within the first 30 days of treatment [25]. This is particularly relevant in routine clinical practice, where adherence and patient comfort are key factors in the management of motor fluctuations.

Inhaled levodopa may be used at any stage of PD, provided that patients can use the device independently (or with caregiver support) and do not have significant pulmonary disease. Nevertheless, identifying the patient profiles most likely to benefit from treatment remains particularly important. In this study, similar patient-reported response rates were observed across all analyzed subgroups. Furthermore, the perceived efficacy and ease of use were consistent among patients with cognitive impairment and sedentary lifestyles, suggesting that the treatment may be suitable for different clinical profiles in real-world practice. These observations highlight the importance of personalizing the administration strategy to the patient’s characteristics and routines, adjusting both the dose and modality (on-demand or scheduled) to optimize the patient experience. However, given the limited sample size of each subgroup, these results should be interpreted with caution and require confirmation in adequately powered studies.

This study has several limitations that should be considered. Firstly, it was an observational, cross-sectional study with a small sample size limiting the generalizability of the findings and with insufficient statistical power to detect meaningful differences between patient subgroups. Because the study included only patients with sufficient experience using inhaled levodopa to complete the questionnaires, patients who never used the treatment, were unable to use the inhaler successfully, or could not reliably recall their experience were not represented. Consequently, patient satisfaction and perceived effectiveness may have been overestimated. Secondly, efficacy and treatment satisfaction were assessed exclusively through patient-reported outcomes collected during a single study visit rather than objective clinical measures and are therefore subject to recall and reporting bias. Thirdly, the study-specific questionnaire used to assess treatment experience was not formally validated, which may have affected the validity and reliability of the collected data. Finally, variability in inhaled levodopa administration regimens may have influenced patients’ perception of efficacy and its impact on activities of daily living. Nevertheless, these findings complement information from clinical trials by reflecting how inhaled levodopa is incorporated into routine clinical practice and how it is perceived by patients in real-world settings.

## 5. Conclusions

This descriptive real-world study reports the clinical experience and satisfaction with inhaled levodopa in routine clinical practice. The results show high patient-reported response rates and satisfaction across different patient profiles, together with good tolerability. Further prospective studies using validated instruments and objective clinical measures are warranted to confirm these results.

## Figures and Tables

**Table 1:** Demographic and clinic characteristics of patients.

Variable	N = 35
Age (years), mean (SD)	70.6 (10.9)
Median (Min; Max)	72.0 (48.0; 88.0)
Gender, n (%)	
Men	17 (48.6%)
Women	18 (51.4%)
PD evolution (year), mean (SD)	10.3 (3.9)
Median (Min; Max)	9.0 (5.0; 22.0)
MNCD, n (%)	
Stage 2	12 (34.3%)
Stage 3	17 (48.6%)
Stage 4	4 (11.4%)
Stage 5	2 (5.7%)
Fluctuations evolution (year), mean (SD)	3.9 (2.1)
Median (Min; Max)	4.0 (1.0; 10.0)
Fluctuations severity, n (%)	
Mild	2 (5.7%)
Moderate	27 (77.1%)
Severe	6 (17.1%)
LEDD (mg), mean (SD)	1020.4 (340.1)
Median (Min; Max)	980.0 (150.0; 1840.0)
Indication for inhaled levodopa ^1^, n (%)	
End-of-dose deterioration	24 (68.6%)
Morning akinesia	20 (57.1%)
Erratic offs	5 (14.3%)
Dose failure	4 (11.4%)
Nocturnal akinesia	1 (2.9%)

^1^ Patients could present more than one indication. Data are expressed as mean (standard deviation, SD) and median (Minimum; Maximum), or as number and percentage (n, %). PD, Parkinson disease; MNCD, Motor/Non-Motor/Cognition/Dependency; LEDD, levodopa equivalent daily dose.

**Table 2:** Clinical response associated with inhaled levodopa.

Variable	N = 35
Response, n (%)	28 (80.0%)
Mild	6 (17.1%)
Moderate	11 (31.4%)
Marked	11 (31.4%)
Response duration (min), mean (SD)	122.1 (74.8)
Median (Min; Max)	90.0 (40.0; 240.0)
Until the next scheduled oral levodopa dose, n (%)	16 (45.7%)
Time to response (min), mean (SD)	17.7 (6.8)
Median (Min; Max)	15.0 (5.0; 30.0)

**Table 3:** Adverse events.

Variable	n = 35
Patients with adverse events, n (%)	16 (45.7%)
Adverse events ^1^, N	21
Cough	10
Throat clearing	3
Throat irritation	2
Anxiety	1
Oral ulcers	1
Dyskinesia	1
Black mucus	1
Nightmares	1
Shortness of breath	1

Data are expressed as number of patients and percentage (n, %) and as number of adverse events (N). ^1^ Patients could experience more than one adverse event.

## Data Availability

The data presented in this study are available from the corresponding author upon reasonable request. The data are not publicly available because they contain information derived from patient medical records, and public sharing is restricted due to patient privacy, confidentiality, and ethical requirements.

## Supplementary Materials

The following supporting information can be downloaded at https://www.mdpi.com/article/10.3390/medsci14040446/s1, Table S1. Global treatment experience questionnaire; Table S2. Factors associated with response rate, time to response, duration and level of response with inhaled levodopa.

## Abbreviations

The following abbreviations are used in this manuscript:

ADLs	Activities of daily living
COMT	Catechol-O-methyltransferase
LEDD	Levodopa equivalent daily dose
MAO-B	Monoamine oxidase B
MNCD	Motor/Non-motor/Cognition/Dependency
