# Supplementary material for: Clinical Use Patterns and Patient-Reported Experience with Inhaled Levodopa in Routine Practice: A Two-Center Study
Source: Med Sci (Basel). 2026 Jul 28;14(4):446. doi: 10.3390/medsci14040446 (PMC13515904; doi:10.3390/medsci14040446)
Supplement: Supplementary file 1 [file medsci-14-00446-s001.zip › medsci-4429828-supplementary.pdf]

Supplementary Table S1. Global treatment experience questionnaire

**Efficacy, each item was scored as 1 (Yes) or 0 (No)**
Have you noticed an improvement in symptoms after inhalation (motor symptoms—such as tremor, rigidity, slowness of movement, or difficulty walking—or non-motor symptoms, such as anxiety, pain, or fatigue)?
Do you feel that the effect is rapid?
Do you feel that the dose is sufficient to control symptoms?
Did the effect last until the next medication dose?
**Ease of use, each item was scored as 1 (Yes) or 0 (No)**
Is it easy for you to know when to use an inhaled dose?
Can you administer the dose without assistance?
Do you find it practical/convenient to carry with you?
Were you able to use the inhalation device without difficulties?
**Adverse events, each item was scored as 0 (Yes) or 1 (No)**
Have you experienced respiratory side effects (dyspnea, shortness of breath, cough, or respiratory infections) after using the treatment?
Have you experienced neurological side effects (dyskinesias, hallucinations, or confusion) after using the treatment?
Have you experienced other side effects (dizziness, nausea, allergy, or throat irritation)?
Have these side effects led you to use the treatment less frequently or to stop using it?
**Impact on Activities of Daily Living, each item was scored as 1 (Yes) or 0 (No)**
Do you think you need less assistance since using the treatment?
Do you feel greater freedom of movement thanks to having this treatment available, for example going out more often?
Have you been able to carry out more activities?
Do you feel safer or less anxious knowing that you have this treatment available to control your symptoms?

Supplementary Table S2. Factors associated with response rate, time to response, duration and level of response with inhaled levodopa.

	**With cognitive impairment (n = 20)**		**Without cognitive impairment (n = 15)**	**P-value**
**Response rate**, n (%)	17 (85.0%)		11 (73.3%)	0.393
**Time to response**, min	18.7 (6.8)		16.1 (6.8)	0.344
**Response duration**, min	180.0 (69.3)		93.1 (62.0)	0.052
Determined, n (%)	4 (20.0%)		8 (53.3%)	0.028
Until next dose, n (%)	13 (65.0%)		3 (20.0%)	
**Response level**, n (%)				0.795
Mild	4 (20.0%)		2 (13.3%)	
Moderate	6 (30.0%)		5 (33.3%)	
Marked	7 (35.0%)		4 (26.7%)	
	**Active (n = 18)**		**Sedentary (n = 17)**	
**Response rate**, n (%)	15 (83.3%)		13 (76.5%)	0.612
**Time to response**, min	17.0 (7.0)		18.46 (6.8)	0.580
**Response duration**, min	121.9 (75.4)		122.5 (85.0)	0.428
Determined, n (%)	8 (44.4%)		4 (23.5%)	
Until next dose, n (%)	7 (38.9%)		9 (52.9%)	
**Response level**, n (%)				0.370
Mild	2 (11.1%)		4 (23.5%)	
Moderate	5 (27.8%)		6 (35.3%)	
Marked	8 (44.4%)		3 (17.6%)	
	**Fixed (n = 11)**	**On-demand (n = 18)**	**Combined (n = 6)**	
**Response rate**, n (%)	8 (72.7%)	16 (88.9%)	(88.9%)	0.264
**Time to response**, min	19.7 (6.9)	16.4 (6.8)	18.8 (7.5)	0.278
**Response duration**, min	150.0 (79.4)	117.5 (79.4)	75 (0)	0.56084
Determined, n (%)	3 (27.3%)	8 (44.4%)	1 (16.7%)	0.457
Until next dose, n (%)	5 (45.5%)	8 (44.4%)	3 (50.0%)	
**Response level**, n (%)				0.17
Mild	4 (36.4%)	2 (11.1%)	0	

Moderate	2 (18.2%)	7 (38.9%)	2 (33.3%)	
Marked	2 (18.2%)	7 (38.9%)	2 (33.3%)	
	**Dependent (n = 14)**		**Independent (n = 21)**	
**Response rate**, n (%)	11 (78.6%)		17 (81.0%)	0.863
**Time to response**, min	18.0 (7.3)		17.5 (6.7)	0.867
**Response duration**, min	150.0 (79.4)		112.8 (75.6)	0.482
Determined, n (%)	3 (21.4%)		9 (42.9%)	0.403
Until next dose, n (%)	8 (57.1%)		8 (38.1%)	
**Response level**, n (%)				0.764
Mild	3 (21.4%)		3 (14.3%)	
Moderate	5 (35.7%)		6 (28.6%)	
Marked	3 (21.4%)		8 (38.1%)	
